# Selective recovery of pyrolyzates of biodegradable (PLA, PHBH) and common plastics (HDPE, PP, PS) during co-pyrolysis under slow heating

**DOI:** 10.1038/s41598-024-67330-0

**Published:** 2024-07-16

**Authors:** Wakana Adachi, Shogo Kumagai, Zhuze Shao, Yuko Saito, Toshiaki Yoshioka

**Affiliations:** 1 Graduate School of Environmental Studies, Tohoku University, 6-6-07 Aoba, Aramaki-aza, Aoba-ku, Sendai, Miyagi 980-8579 Japan; 2 Graduate School of Engineering, Tohoku University, 6-6-07 Aoba, Aramaki-aza, Aoba-ku, Sendai, Miyagi 980-8579 Japan

**Keywords:** Biodegradable plastics, Petroleum-based plastics, Co-pyrolysis, Py-GC/MS, Environmental chemistry, Polymer chemistry, Process chemistry

## Abstract

Pyrolytic synergistic interactions, in which the production of pyrolyzates is enhanced or inhibited, commonly occur during the co-pyrolysis of different polymeric materials, such as plastics and biomass. Although these interactions can increase the yield of desired pyrolysis products under controlled degradation conditions, the desired compounds must be separated from complex pyrolyzates and further purified. To balance these dual effects, this study was aimed at examining pyrolytic synergistic interactions during slow heating co-pyrolysis of biodegradable plastics including polylactic acid (PLA) and poly(3-hydroxybutyrate-*co*-3-hydroxyhexaoate) (PHBH) and petroleum-based plastics including high-density polyethylene (HDPE), polypropylene (PP), and polystyrene (PS). Comprehensive investigations based on thermogravimetric analysis, pyrolysis–gas chromatography/mass spectrometry, and evolved gas analysis-mass spectrometry revealed that PLA and PHBH decompose at lower temperatures (273–378 °C) than HDPE, PP, and PS (386–499 °C), with each polymer undergoing independent decomposition without any pyrolytic interactions. Thus, the independent pyrolysis of biodegradable plastics, such as PLA and PHBH, with common plastics, such as HDPE, PP, and PS, can theoretically be realized through temperature control, enabling the selective recovery of their pyrolyzates in different temperature ranges. Thus, pyrolytic approaches can facilitate the treatment of mixed biodegradable and common plastics.

## Introduction

Countries representing more than 80% of the world economy have pledged to achieve carbon neutrality by mid-century^1^. The management of plastic waste is crucial for meeting these targets. In 2019, the total greenhouse gas (GHG) emissions related to the entire lifecycle of petroleum-based plastics accounted for 1.8 billion tons of carbon dioxide equivalent worldwide, constituting 3.7% of the global emissions. These emissions are projected to more than double by 2060, reaching 4.3 billion tons of carbon dioxide equivalent, or 4.5% of global GHG emissions^2,3^. Despite this significant impact, the amount of global plastic waste is continuously increasing, with an alarming value of 400.3 million tons recorded in 2022^4^. In addition, mismanagement of plastic waste is exacerbating ocean pollution, with annual leakage of plastics expected to increase by 50% between 2020 and 2040, reaching 30 million tons, and 300 million tons of plastics projected to leak into the aquatic environment by 2040.

To address this severe issue, the adoption of bio-based plastics is being promoted. These plastics are defined as either fully or partially derived from biological resources^5^. The production of bio-based plastics generally emits fewer greenhouse gases because the biomass can also absorb CO_2_ from the atmosphere during their growth, making them potentially carbon–neutral. European Union (EU) has formulated the EU Plastics Strategy as part of its Circular Economy Action Plan, focusing on the sourcing, labeling, and use of bio-based and biodegradable composable plastics. The United States is adopting a whole-of-government approach to implement various actions and plans, including raising/consumer awareness of bioplastics and introducing a Certified Bio-based Product label to facilitate the identification and promote the purchase of bioproducts^6^. Japan has formulated a plastic material cycle strategy, aiming the introduce 2 million tons of bio-based plastics by 2030^7^. The global production capacity for bio-based plastics is expected to increase from 1.8 million tons in 2022 to 7.4 million tons in 2028^8^. Additionally, bio-based plastics can be categorized into non-biodegradable and biodegradable plastics. The bio-based/non-biodegradable plastics are chemically identical to their petroleum counterparts and are designed to degrade into water and carbon dioxide under certain conditions. Representative examples of bio-based/biodegradable plastics with significant potential include polylactic acid (PLA) and polyhydroxyalkanoates (PHA), expected to account for 31.0% and 4.8% of the global production capacities of bioplastics in 2023, respectively, with proportions increasing to 43.6% and 13.5% in 2028^9^. PLA and PHA are already being used in various products, and the products are treated in a similar manner as products derived from petroleum-based plastics during and after use. This aspect highlights the potential of co-processing biodegradable and petroleum-based plastics.

Although the bio-based/biodegradable plastics are considered to be eco-friendly alternative to common plastics, they should not be thrown away casually without considering their specific properties and disposal requirements. Some of these plastics are compostable, such as PLA^10^, however, their degradation typically requires industrial composting facilities, which are very slow under natural conditions in soil or marine^11,12^. In addition to accumulation, the microplastics produced by bio-based/biodegradable plastics in the environment cannot be ignored, and they can accumulate in ecosystem, which in turn bioaccumulate in the bodies of humans and other organisms^13,14^. An increasing number of researchers are realizing that sustainable disposal strategies are indispensable for bio-based/biodegradable plastic wastes^15,16^.

Pyrolysis is a method used to convert polymers into chemical feedstock under inert atmospheric conditions. At high temperatures, several chemical bonds in various polymers are cleaved into smaller molecules, with value equivalent to those of native molecules. Various researchers have explored the effective recycling of waste plastics through pyrolysis^17,18^. By controlling various parameters, such as pyrolysis temperature, heating rate, pressure, and process time, the product yield can be increased^19^. Pyrolysis also offers advantages over mechanical recycling as it can treat various types of plastics. Kumagai and Yoshioka^20,21^ have highlighted the possibility of treating hard-to-recycle plastics such as polyethylene terephthalate, polyvinyl chloride, and polyurethane through pyrolysis. Additionally, through a lifecycle assessment, Andooz et al.^22^ have suggested that pyrolysis has a lower global warming potential compared with other waste plastic treatment methods. Jeswani et al.^23^ conducted a lifecycle assessment to compare pyrolysis with energy recovery and mechanical recycling techniques. The authors reported that the conversion of plastic waste into naphtha-cracker feedstock through pyrolysis has a nearly 50% lower climate change impact and lifecycle energy use compared with energy recovery. Thus, pyrolysis represents a promising recycling technique for waste plastics.

In addition to common plastics, the pyrolysis of biodegradable plastics has been explored. Various studies have highlighted that the dominant reaction pathway of PLA involves intramolecular transesterification, resulting in cyclic oligomers^24^. For poly(*β*-hydroxybutyric acid) (PHB) pyrolysis, the dominant reaction pathway is stereoselective *cis*-elimination, yielding crotonic acid and its oligomers^25^. Shao et al.^26^ examined the pyrolysis behavior of PLA and poly-3-hydroxybutyrate-co-3-hydroxyhexanoate (PHBH) with various temperatures and heating rates and demonstrated that the highest yields of lactide and crotonic acid, high-value chemicals from PLA and PHBH, are obtained at 400 and 600 °C, respectively. Saeaung et al.^27^ investigated the catalytic and non-catalytic pyrolysis of PLA and reported that 79% lactide can be extracted from PLA through catalytic pyrolysis with zeolite at 400 °C.

Separation of mixed plastic waste poses significant challenges, often proving to be both difficult and uneconomical^28,29^. In response to this, the opportunities for co-processing biodegradable and common plastics are steadily increasing. Co-pyrolysis, a promising approach in this domain, not only addresses the complexities of mixed plastic waste but also facilitates the emergence of pyrolytic synergistic interactions. However, existing research predominantly focused on enhancing the yield of specific compounds through co-pyrolysis, with ongoing investigations into the influence of biomass additives and various co-pyrolysis conditions. Sun et al.^30^ reported that the presence of biomass additives, such as cellulose, hemicellulose, and lignin, can enhance the lactide yield and decrease the required energy. Kasataka et al.^31^ clarified that the pyrolysis of ceder wood (CW) dispersed in polyethylene (PE) melt inhibits char formation and promotes the radical interaction between the CW and PE pyrolyzates and hydrocarbon production from PE. Xie et al.^32^ introduced the response surface methodology to predict the pyrolyzate yields affected by synergies during the co-pyrolysis of cellulose and PE, demonstrating that co-pyrolysis can promote C_5_–C_28_ hydrocarbon formation with increasing temperature and enhanced liquefaction.

However, research specifically targeting the co-pyrolysis of biodegradable and common plastics is limited. Omura et al.^33^ examined the selective recovery of lactide from a mixture of PLA and linear LDPE (LLDPE) through catalytic pyrolysis at temperatures below 360 °C using magnesium oxide (MgO). They concluded that even when PLA forms a blend with LLDPE, LLDPE does not influence the feedstock recycling of PLA. Miskolczi et al.^34^ conducted co-pyrolysis of HDPE and PLA using a batch reactor at 400–500 °C to explore the treatment of mixed waste materials. However, they did not observe any clear synergistic effects. Wu et al.^35^ observed that the co-pyrolysis of PLA/ABS blends occurs more rapidly than the pyrolysis of PLA or ABS individually. While these studies have examined the selective recovery of certain compounds from mixed plastic streams, the broader understanding of pyrolytic interactions during co-pyrolysis remains inadequate. There remains a notable gap in the understanding of the intricate pyrolytic interactions between these materials.

Addressing this knowledge gap is imperative for the advancement of waste recycling strategies. Therefore, this study aims to shed light on the impact of integrating biodegradable plastics into the pyrolysis recycling process of conventional plastics, thereby contributing to the development of more efficient and sustainable recycling methods. Elucidating these interactions is vital for effectively recovering chemical feedstocks from mixed wastes. Considering these aspects, in this study, PLA and PHBH were selected as representative biodegradable plastics, while HDPE, PP, and polystyrene (PS) were selected as common petroleum-based plastics. This selection was based on their significant production volumes, as PE, PP, and PS constitute approximately 33.8%, 24.4%, and 12.2% of total waste plastics in Japan^36^. Given the distinct chemical structures of these plastics, the investigation of their pyrolytic interactions holds significant potential. The pyrolysis characteristics of biodegradable plastics, common plastics, and their mixtures were investigated through thermogravimetric analysis (TGA), evolved gas analysis-mass spectrometry (EGA-MS), and pyrolysis–gas chromatography/mass spectrometry GC/MS (Py-GC/MS).

## Materials and methods

### Materials

PLA was purchased from Standard Test piece Co., Ltd. (Kanagawa, Japan). PHBH was supplied by Kaneka Co., Ltd. (Tokyo, Japan). Virgin-grade HDPE (*M*_w_ = 150,000), PP (*M*_w_ = 200,000) and PS (*M*_w_ = 350,000, Sigma-Aldrich, USA) were used. The samples did not contain any additives. Before experimentation, samples were ground at − 196 °C using a ball cryomill (CryoMill, Retsch Co., Ltd., Germany), and sieved to achieve a uniform particle size (104–150 μm) using a sieve shaker (AS200, Retsch Co., Ltd., Germany). The various samples were then gently stirred in a platinum sample cup using a stainless steel rod to ensure thorough mixing for even distribution. Because only 1.0 or 10.0 mg samples were required in the pyrolysis tests, the desired ratio of mixed samples was directly achieved in the sample cup using ultra-accurate measurements (MCA2.7SM-S01, *d* = 0.1 µg, Max2100mg, Sartorius Co., Ltd., Germany).

### TGA

TGA was performed using a thermogravimetric analyzer (STA7200RV, Hitachi High-Tech Science Corporation, Japan). Specifically, 10 mg of a sample was heated from 50 to 700 °C at a heating rate of 10 °C/min under a nitrogen atmosphere with a flow rate of 200 mL/min. The TGA curve was obtained using Eq. (1):

1 $${W}_{\text{cal}, T} \left(wt\%\right)={R}_{\text{B.P}.}\times {W}_{\text{B.P}, T}+{R}_{\text{C.P}.}\times {W}_{\text{C.P}, T}$$

where ${W}_{\text{cal}, T}$ is the calculated thermogravimetric (TG) or derivative thermogravimetric (DTG) value at *T* [°C]; ${R}_{\text{B.P}}$ and ${R}_{\text{C.P}}$ denote the weight ratios of biodegradable plastic or common plastic in the mixed sample, respectively; and ${W}_{\text{B.P}, T}$ and ${W}_{\text{C.P}, T}$ denote the experimental values of biodegradable plastic and common plastic at *T* [°C], respectively.

This analysis was conducted for each plastic and mixtures of biodegradable and common plastics at different weight ratios (biodegradable plastics:common plastics weight ratio = 100:0, 50:50, 20:80, 10:90, and 0:100). Owing to space restrictions, only the results for the 50:50 ratio are presented herein, and the other results can be found in the Supporting Information (SI).

### Py-GC/MS

To identify pyrolyzates, a multi-shot pyrolyzer (EGA/PY-3030D, Frontier Laboratories Ltd., Japan) combined with a GC/MS (GC: 7890, MS: 5975, Agilent, USA) was used. An Ultra Alloy® Capillary Column UA^+^-5 (30 m length, 0.25 mm i.d., 0.25 μm film thickness with 95% polydimethylsiloxane and 5% poly diphenyl dimethyl siloxane stationary phase, Frontier Laboratories Ltd., Japan) was used, with helium used as the carrier gas at a flow rate of 1 mL/min. The pyrolyzer was heated from 50 to 700 °C at a rate of 10 °C/min. Each sample (1 mg) was loaded, and the pyrolysis products were trapped by a cryotrap until the pyrolyzer temperature reached the set temperature. Subsequently, the cryotrap was removed, and the products were transferred to the MS through the separation column. The inlet mode was set to a split mode with a split ratio of 100:1, and the inlet temperature was 300 °C. The GC oven program was set as 40 °C (5 min) → 20 °C/min → 300 °C (10 min). The mass spectrometer operated in the scan mode, with a scanning range of *m*/*z* 10–600 at 70 eV. The MS transfer line temperature, source temperature of the mass selective detector (MSD), and MS quadrupole temperature were set as 280, 230, and 150 °C, respectively. An MSD ChemStation (version F.01.03.2357, Agilent, USA) software with NIST17 library was installed to calculate the peak area values. Variations in biodegradable plastics:common plastics weight ratios greater than 50:50 were not pursued in Py-GC/MS analysis due to observed differences in compound response, particularly the significantly higher response of compounds from common plastic compared to biodegradable plastics.

### EGA-MS

EGA-MS analysis was conducted to investigate the evolution behavior of each pyrolyzate. The pyrolyzer conditions were the same as those used for the Py-GC/MS analysis. Approximately 1 mg samples were placed into the sample cup, and heated from 50 to 700 °C at a rate of 10 °C/min. The pyrolyzates were transferred through an Ultra Alloy® deactivated metal capillary tube UADTM (2.5 m length, 0.15 mm i.d., 0.47 mm o.d., Frontier Laboratories Ltd., Japan) column to the MS without separation. The GC oven temperature was maintained at 300 °C. The weight ratios of mixed samples were the same as those used in TGA (i.e., biodegradable plastics:common plastics = 100:0, 50:50, 20:80, 10:90, and 0:100).

## Results and discussion

### Pyrolysis behavior

**Figure 1:**
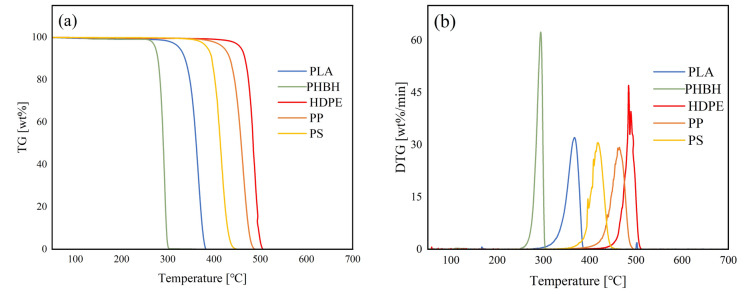
(**a**) TG and (**b**) DTG curves of each plastic.

Figure 1 shows the weight loss behavior of each plastic. The start and end temperatures were defined by the DTG value of 5 wt.%/min. The temperature ranges varied among the plastic samples, with biodegradable plastics exhibiting lower ranges than those of common plastics. Among the common plastics, HDPE and PS exhibited the highest (458–499 °C) and lowest (386–444 °C) temperature ranges, respectively, with the range of PP being 422–478 °C at a heating rate of 10 °C/min. In comparison, PLA and PHBH exhibited lower temperature ranges of 325–378 and 273–298 °C, respectively. Table S1 outlines the start, end, and peak-top temperatures of all samples. All plastics exhibited one-step weight loss without any residue production. Notably, Das and Tiwari^37^ reported the degradation temperatures of HDPE (433–493 °C), PP (409–469 °C), and PLA (323–374 °C) at a heating rate of 10 °C/min. Saad et al.^38^ indicated that the pyrolysis temperature ranges of HDPE, PP, and PS are 464–497, 441–483, and 420–452 °C, respectively, at a heating rate of 10 °C/min. Kopinke et al.^24^ reported that PLA degradation initiates at 225 °C and ends at 370 °C, while PHB degradation initiates at 255 °C and ends at 305 °C under a heating rate of 5 °C/min. Thus, the pyrolysis temperatures observed in this work were consistent with these reports, validating the TG measurements.

**Figure 2:**
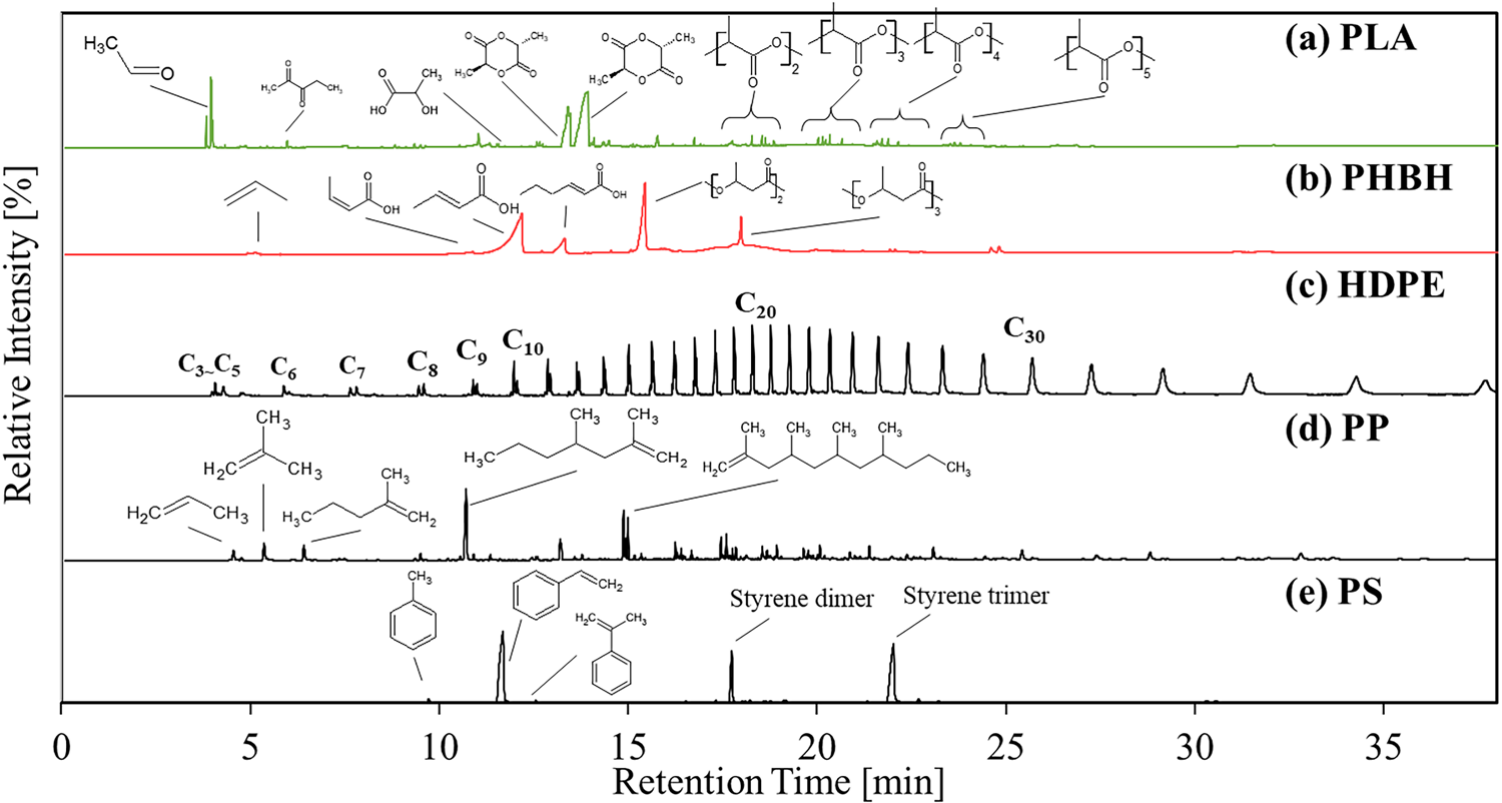
Pyrograms of (**a**) PLA, (**b**) PHBH, (**c**) HDPE, (**d**) PP, and (**e**) PS.

As shown in Fig. 2a, the main pyrolyzates of PLA were acetaldehyde; *D-, L-*, and *meso*-lactides; and their oligomers. *Meso*-lactide is typically produced through a free radical reaction, and *D-* and *L-*lactides are produced by transesterification reactions^24,39^. The produced oligomers were considered cyclic lactide, with reference to the works of Shao et al. ^26^ and Arrieta et al.^40^. The main pyrolyzates of PHBH were propylene, isocrotonic acid, crotonic acid, 2-hexenoic acid, and their dimers (Fig. 2b). HDPE yielded aliphatic hydrocarbons with a wide range of carbon numbers through random radical scission. Under the current analytical conditions, C_1_–C_35_ hydrocarbons were observed (Fig. 2c). PP pyrolyzates were more complex than those of HDPE because of the presence of a methyl unit. Through a comparison with the NIST17 library, propylene, pentane, 2-methyl-1-pentene, and 2,4-dimethyl 1-heptene were identified, with a matching score exceeding 90% (Fig. 2d). Moreover, with reference to the Py-GC/MS Data Book of Synthetic Polymers^41^, propylene, n-pentane, 2-,ethyl-1-pentene, 2,4-dimethyl-1-pentene, and propylene oligomers with various lengths were identified. The main PS pyrolyzates were styrene, styrene dimers, and styrene trimers (Fig. 2e). The pyrogram pattern was consistent with that reported in the Data Book^41^. The volatile emission behavior of each sample was investigated through EGA-MS analysis, and the peak-top temperatures of total ion chromatograms (TICs) obtained from each sample are summarized in Table S2. These temperatures were slightly lower than the DTG peak-top temperatures (Table S1) owing to the superior heat conductivity in the Py-GC/MS method attributable to the smaller amount of sample loading. Details of the evolution behavior of selected compounds are presented in the following sections.

### Co-pyrolysis of PLA and common plastics

**Figure 3:**
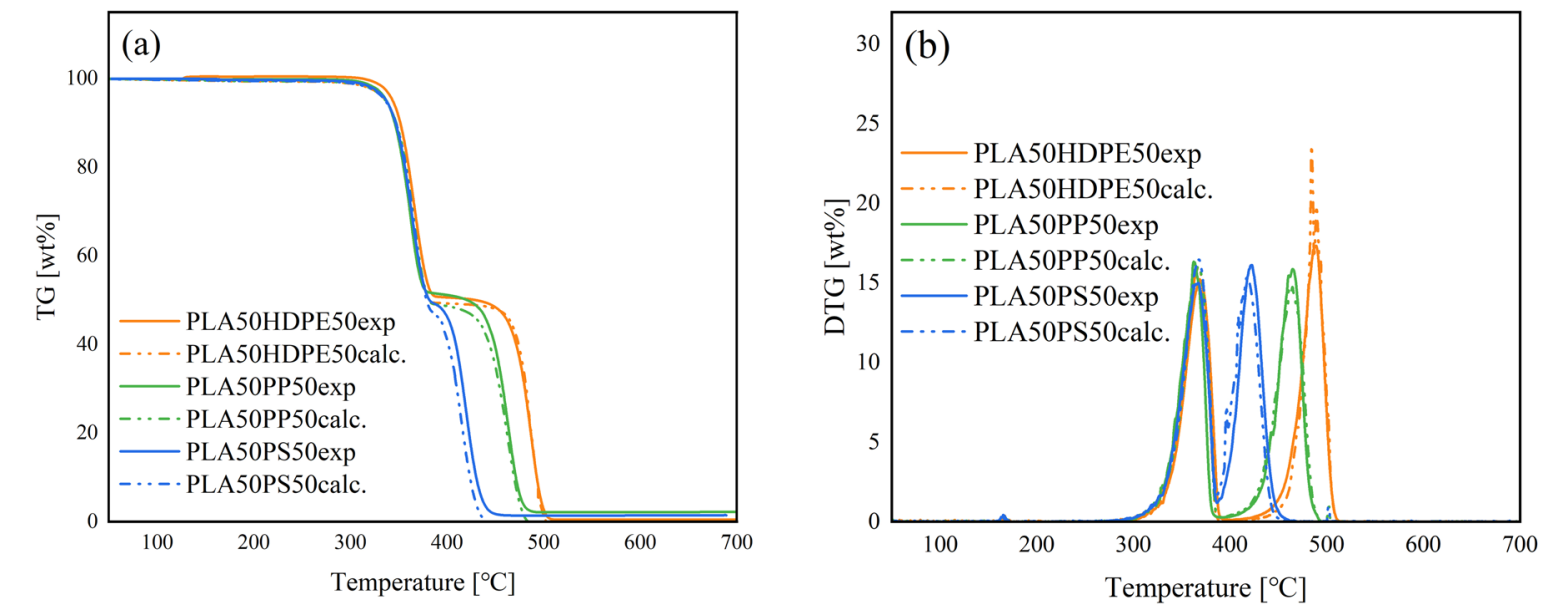
(**a**) TG and (**b**) DTG curves of PLA50HDPE50, PLA50PP50, and PLA50PS50.

**Figure 4:**
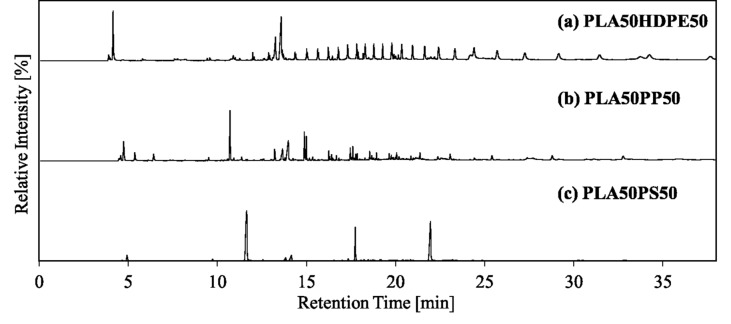
Pyrograms of (**a**) PLA50HDPE50 (PLA:HDPE = 50:50), (**b**) PLA50PP50 (PLA:PP = 50:50), and (**c**) PLA50PS50 (PLA:PS = 50:50).

**Figure 5:**
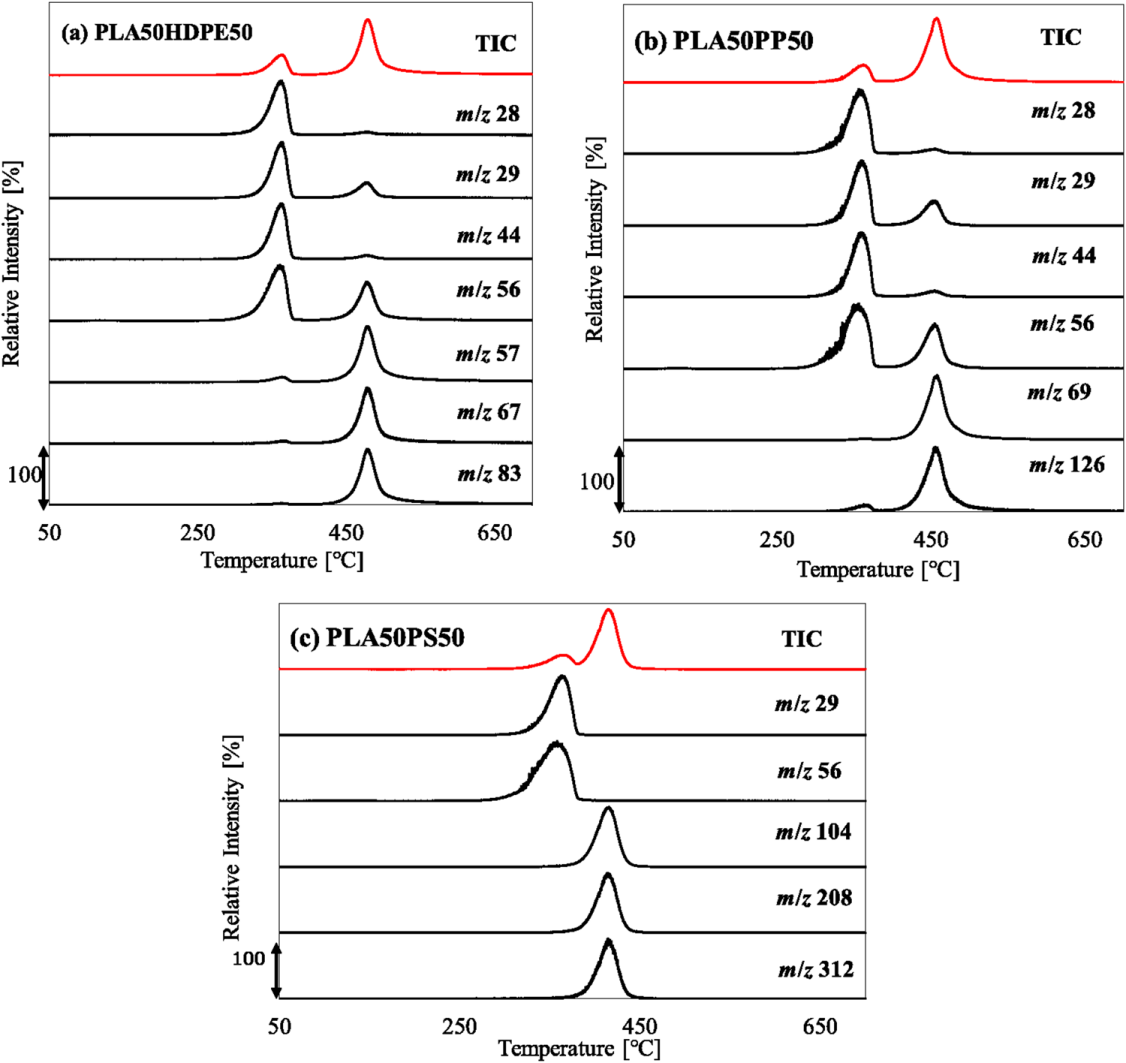
TICs and EICs of (**a**) PLA50HDPE50, (**b**) PLA50PP50, and (**c**) PLA50PS50, obtained by EGA-MS analysis.

The co-pyrolysis behavior of PLA and common plastics with a mixing ratio of 50:50 was analyzed by TGA (Fig. 3). The solid and dotted lines show the experimental curves and calculated curves (using Eq. (1)), respectively. PLA50HDPE50 exhibited a two-step weight loss with temperature ranges of 315–389 and 434–509 °C, consistent with those of PLA and HDPE (Fig. 1). The experimental TG/DTG curves exhibited a good fit with their calculated curves. Similar trends were observed for other mixing ratios (PLA:HDPE = 20:80 and 10:90), as shown in Figures S1 and S2. Thus, no pyrolytic synergistic interactions were identified through the weight loss behavior. Py-GC/MS revealed the type of pyrolyzates obtained by co-pyrolysis of PLA50HDPE50 (Fig. 4a). *Meso*-lactide; *D-, L*-lactides; and their oligomers were obtained from PLA, while HDPE produced hydrocarbons with different carbon numbers. No new compounds were observed that were not obtained from PLA and HDPE. Figure 5a shows the TIC and extracted ion chromatograms (EICs) obtained by the EGA-MS analysis of PLA50HDPE50. Results for other mixing ratios can be found in Figure S5. The peak-top temperatures for the first and second gas evolution were 357–363 and 476–479 °C, respectively, consistent with those of PLA and HDPE (Table S2).

To examine the evolution behavior of the main pyrolyzates from PLA, specific ions for selected for monitoring: *m*/*z* 28 (molecular ion of carbon monoxide), *m*/*z* 29 (main fragment ion of acetaldehyde), *m*/*z* 44 (molecular ion of carbon dioxide), and *m*/*z* 56 (main fragment ion of lactides). For HDPE, *m*/*z* 57, 67, and 83 (fragment ions of hydrocarbons, i.e., C_4_H_9_^+^, C_5_H_7_^+^, and C_6_H_11_^+^, respectively) were monitored. Notably, the ions (*m*/*z* 29 and 56) selected for PLA pyrolyzates were also produced from HDPE pyrolyzates. Moreover, the hydrocarbons selected from HDPE (*m*/*z* 57) were also produced by PLA. The peak-top temperatures of the selected ions were consistent with those observed in the TIC. Thus, no pyrolytic synergistic interactions occurred during the EGA-MS analysis. In conclusion, PLA and HDPE independently decomposed under this slow heating condition and did not influence the pyrolysis behavior.

During the co-pyrolysis of PLA/PP, a two-step weight loss similar to that for PLA/HDPE co-pyrolysis was observed. However, PP degradation occurred at a lower temperature range (410–489 °C) compared with that of HDPE (Fig. 1). Nevertheless, the PLA pyrolysis temperature during the co-pyrolysis of PLA50PP50 was similar to that for PLA50HDPE50. The calculated TG/DTG curves were consistent with the experimental curves. The same trends were observed for PLA20PP80 and PLA10PP90. According to Fig. 4b, i.e., the pyrogram of PLA50PP50, both PLA and PP pyrolyzates were observed, with no new compounds observed during the co-pyrolysis of PLA and PP. Figure 5b shows the evolved gas profiles during co-pyrolysis of PLA50PP50. The peak-top temperatures of the first and second peaks were the same as those obtained from the pyrolysis of PLA and PP. The same ions (*m*/*z* 28, 29, 44, and 56) from PLA were monitored, while ions *m*/*z* 29 and 56 were also produced from PP pyrolyzates including 2,4 dimethyl-1-heptene and 2-methyl-1-petene. The selected ion *m*/*z* 69 was a fragment ion of hydrocarbons derived from PP, and *m*/*z* 126 corresponded to the molecular ion of 2,4-dimethyl-1-heptene. The peak-top temperatures from the TIC and selected EICs were the same as those observed in the pyrolysis of PLA and PP. Thus, no pyrolytic synergistic interaction was identified during EGA-MS analysis. In conclusion, PLA and PP independently decomposed under this slow heating condition and did not influence the pyrolysis behavior.

Because the PS degradation temperature was lower than those of HDPE and PP, the temperature range of PS and PLA is the closest combination in this study. Although PLA50HDPE50 and PLA50PP50 exhibited clear two-step weight loss, PLA50PS50 displayed a slightly overlapped weight loss region (Fig. 1), with the first-step weight loss initiating from 311 °C and the second-step weight loss terminating at 451 °C. Regardless of this overlapped weight loss region, the experimental TG/DTG curves fit the calculated curves. Thus, no pyrolytic interaction between PLA and PS was observed through TGA. The pyrogram of PLA50PS50 is shown in Fig. 4c. Both PLA and PS pyrolyzates were identified, and no compound was newly produced during the co-pyrolysis of PLA50PS50. Figure 5c shows the EGA-MS profiles of PLA50PS50. Consistent with TGA, a two-step gas evolution was observed (with the first and second steps corresponding to PLA and PS, respectively). The temperature ranges of the two peaks, 363–370 and 413–414 °C, were similar to those obtained from PLA and PS pyrolysis. The extracted ions of *m*/*z* 104, 208, and 312 corresponded to molecular ions of styrene, styrene dimer, and styrene trimer, respectively. As in the case of the co-pyrolysis of PLA/HDPE and PLA/PP, the peak-top temperatures of the selected ions from PLA (*m*/*z* 28, 29, 44, and 56) and PS (*m*/*z* 104, 208, and 312) were identical to those obtained from PLA and PS pyrolysis. Thus, no pyrolytic interaction occurred between PLA and PS during co-pyrolysis under the current heating method.

### Co-pyrolysis with PHBH

**Figure 6:**
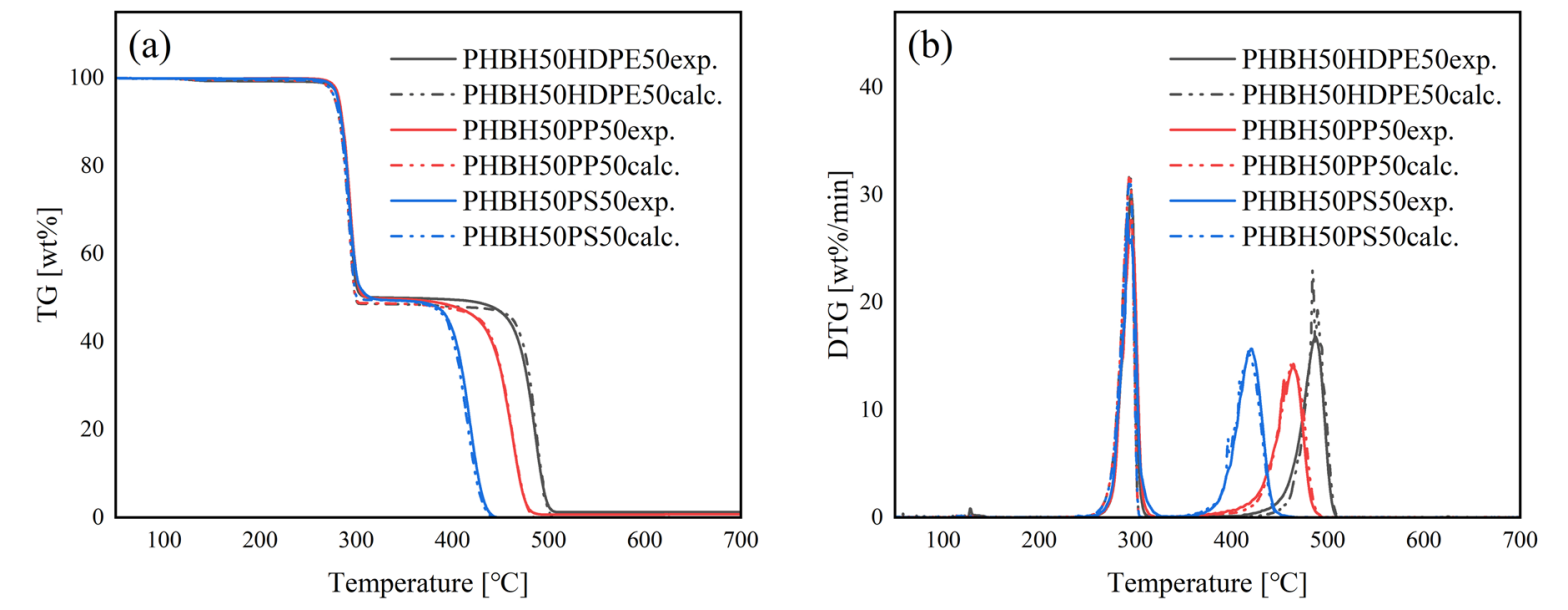
(**a**) TG and (**b**) DTG curves of PHBH50HDPE50, PHBH50PP50, and PHBH50PS50.

**Figure 7:**
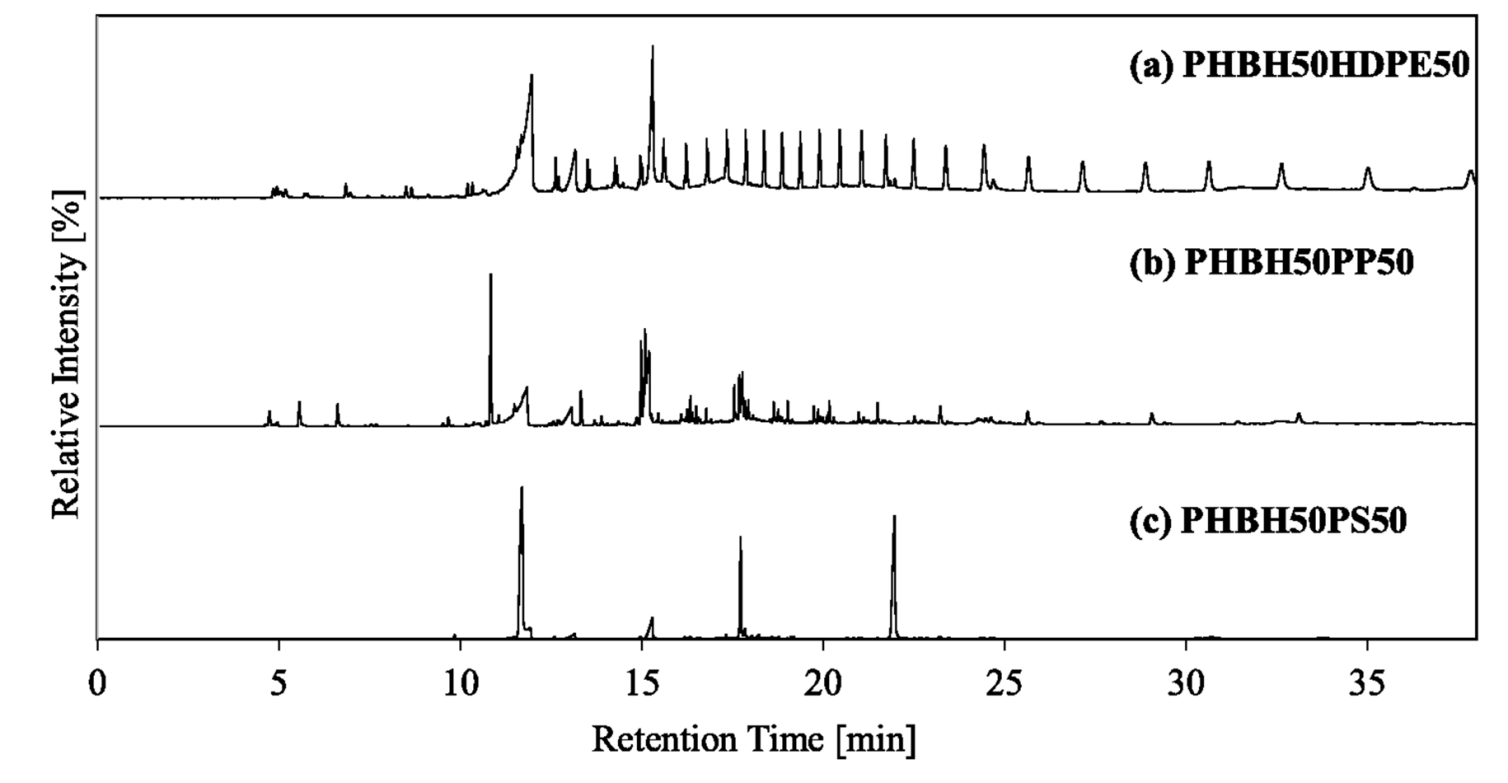
Pyrograms of (**a**) PHBH50HDPE50 (PHBH:HDPE = 50:50), (**b**) PHBH50PP50 (PHBH:PP = 50:50), and (**c**) PHBH50PS50 (PHBH:PS = 50:50).

**Figure 8:**
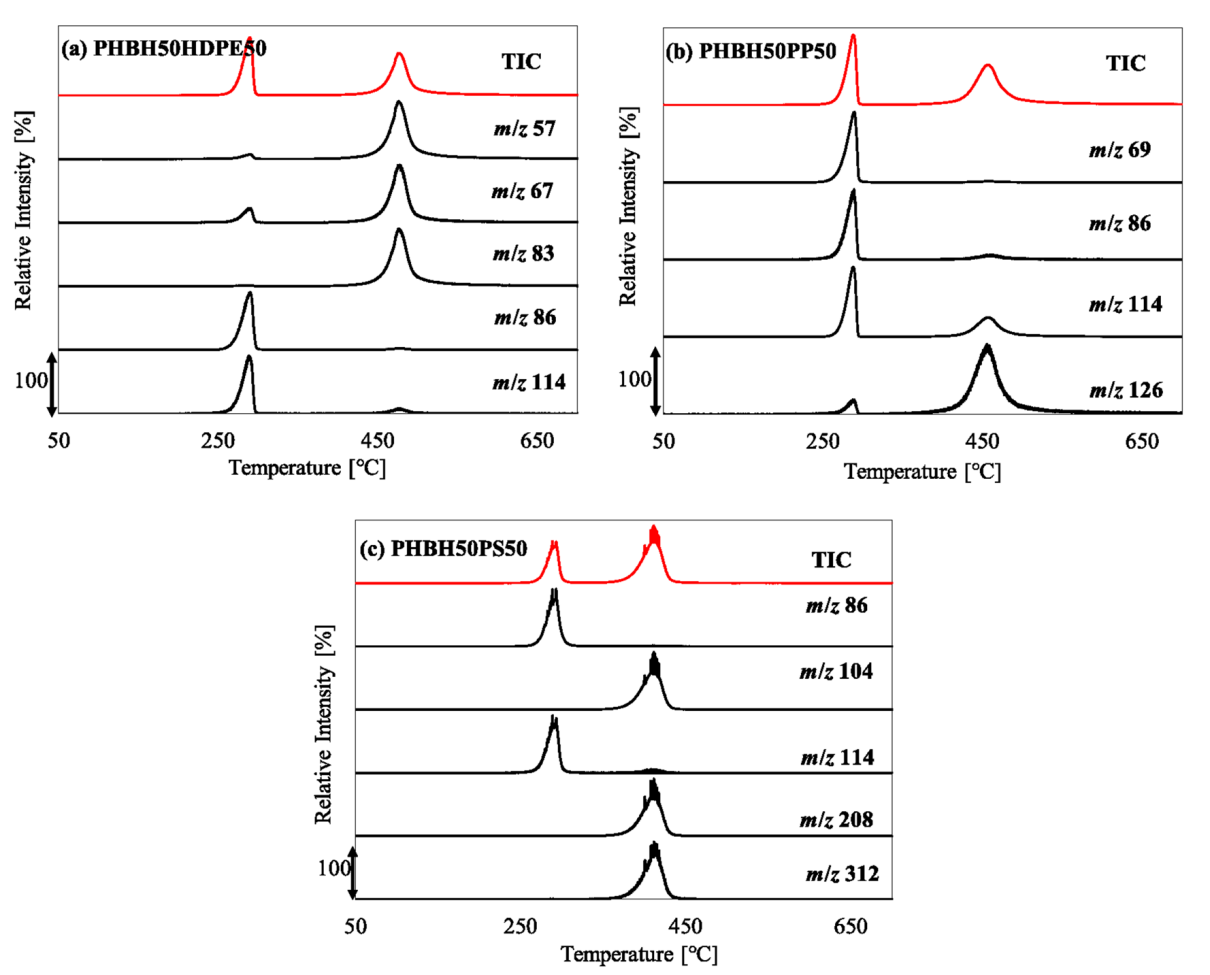
Evolved gas profiles of (**a**) PHBH50HDPE50, (**b**) PHBH50PP50, and (**c**) PHBH50PS50.

During the co-pyrolysis of PHBH/HDPE, a two-step weight loss behavior was observed by TGA (Fig. 6). The first weight loss (267–319 °C) corresponded to PHBH degradation, while the second (428–507 °C) pertained to HDPE degradation. The experimental and calculated curves exhibited a good fit, indicating no pyrolytic synergistic interaction under this condition. The pyrogram of PHBH50HDPE50 is shown in Fig. 7a. Both PHBH and HDPE pyrolyzates were observed, while no new compound was produced during the co-pyrolysis of PHBH/HDPE. The EGA-MS results for PHBH50HDPE50 are shown in Fig. 8a. In addition to PHBH50HDPE50, PHBH20HDPE80 and PHBH10HDPE90 showed two-step gas evolution (Figure S8). The peak-top temperatures of the first and second gas evolution were 289–290 and 477–479 °C. The extracted ions of *m*/*z* 86 and 114 from PHBH corresponded to the molecular ions of (iso)crotonic acid and 2-hexenoic acid, respectively. Notably, *m*/*z* 114 was a fragment ion of hydrocarbons (C_8_H_18_^+^) produced from HDPE. The extracted ions, *m*/*z* 57 (C_4_H_9_^+^), 67 (C_5_H_7_^+^), and 83 (C_6_H_11_^+^), were representatives fragment ions of hydrocarbons derived from HDPE, whereas *m*/*z* 57 was a fragment ion of crotonic acid and 2-hexenoic acid derived from PHBH. The fragment ion behavior demonstrated that no pyrolytic interactions occurred during this co-pyrolysis under the selected conditions.

The co-pyrolysis of PHBH/PP and PHBH/PS showed the same trends as those for PHBH/HDPE. The two-step weight loss corresponded to PHBH degradation and PP or PS degradation, and the experimental and calculated curves fit well. No new compound was produced during the co-pyrolysis of PHBH50PP50 and PHBH50PS50, as indicated by the pyrograms of PHBH50PP50 (Fig. 7b) and PHBH50PS50 (Fig. 7c). Additionally, the EGA-MS profiles of the TICs for PHBH50PP50 (Fig. 8b) and PHBH50PS50 (Fig. 8c) showed two-step gas evolution corresponding to PHBH degradation and PP or PS degradation, with temperatures consistent with those observed in the TGA. Moreover, the temperatures were identical, as confirmed by neat pyrolysis of PHBH, PP, and PS. The emission temperatures of the selected ions, *m*/*z* 86 and 114 for PHBH; *m*/*z* 69 and 126 for PP; and *m*/*z* 104, 208, and 312 for PS, were the same as those observed in the neat pyrolysis of PHBH, PP, and PS. Thus, no pyrolytic interactions occurred during the co-pyrolysis of PHBH/PP and PHBH/PS under the current conditions.

## Conclusions and perspectives

Biodegradable plastics are being widely used in containers, packaging, cutlery, and sheets for agriculture and horticulture^9^ by substituting common plastics. Therefore, mixtures of biodegradable and common plastics are being commonly generated, and effective strategies to recycle these mixtures must be identified.

**Figure 9:**
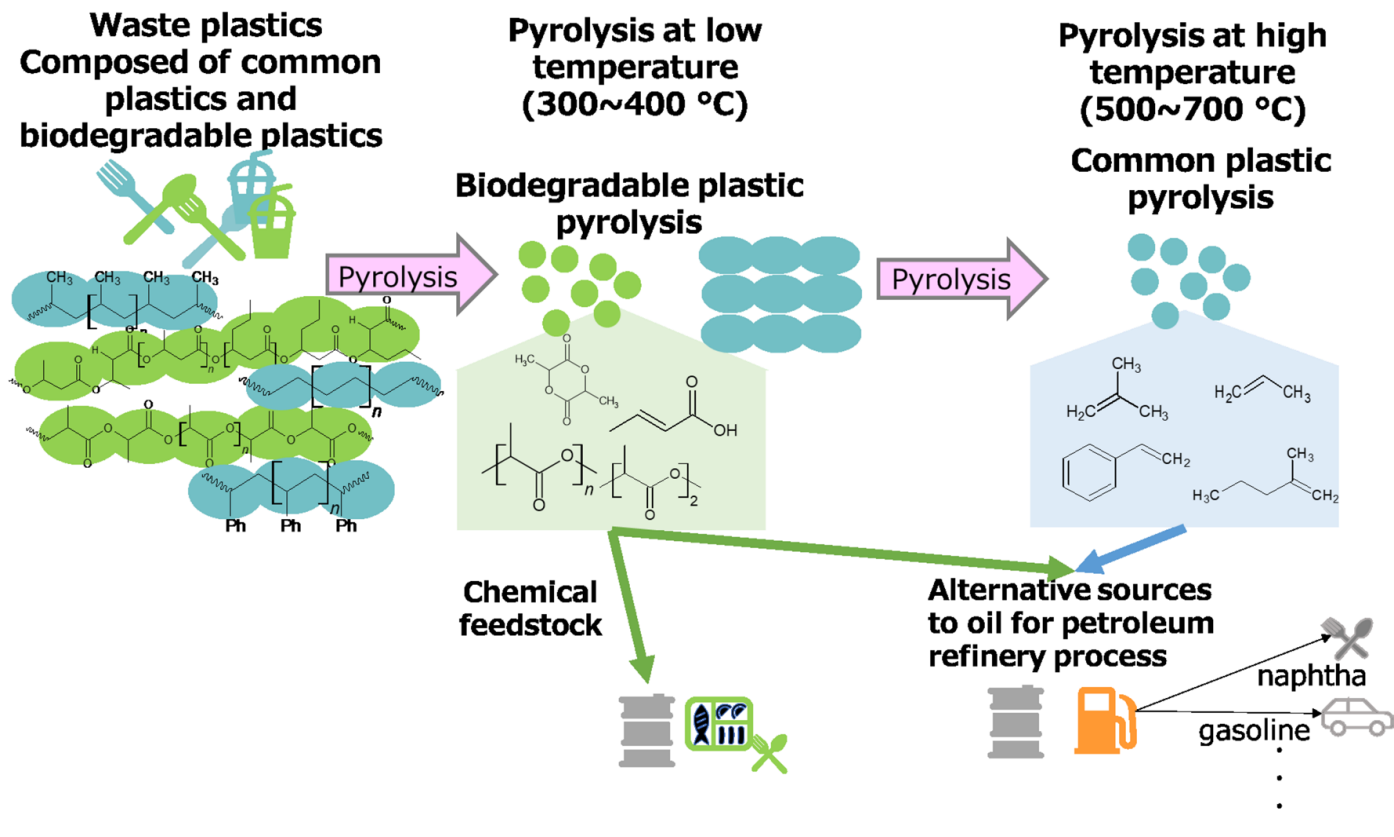
Schematic of feedstock recovery from a mixture of biodegradable and common plastics.

The degradation temperature ranges of PLA (325–378 °C) and PHBH (273–298 °C) were lower than those of HDPE (458–499 °C), PP (422–478 °C), and PS (386–444 °C). TGA, EGA-MS, and Py-GC/MS investigations revealed that each polymer independently decomposed without pyrolytic interactions. This independent degradation offers a significant advantage for recovering chemical feedstock from a mixture of biodegradable plastics (PLA or PHBH) and common plastics (HDPE, PP, PS). Figure 9 illustrates feedstock recovery from their mixtures. In the first step, the biodegradable plastics were pyrolyzed at lower temperatures. The main pyrolyzates of PLA were lactides and their oligomers, which are sources for PLA synthesis. Another major pyrolyzate was acetaldehyde, which is a source for several acids and aldehydes. Crotonic acid, 2-hexenoic acid, and their oligomers were the main pyrolyzates from PHBH, representing monomers of several co-polymers. The market for these chemicals obtained from PLA and PHBH pyrolysis is continuously expanding. Moreover, hydrocarbons produced from HDPE, PP, and PS can be utilized in the petroleum industry.

Overall, our findings demonstrate the absence of pyrolytic interactions between PLA/PHBH and HDPE/PP/PS during their co-pyrolysis and the ability of each polymer to undergo independent pyrolysis during slow-heating co-pyrolysis. The lack of interaction is crucial as it implies that the existing recycling infrastructure for common plastics remains largely unaffected by the inclusion of biodegradable plastics. By controlling the pyrolysis temperature, the separation of products from different plastics can be readily achieved. These insights are valuable for identifying future strategies for the recycling of biodegradable and common plastics, ensuring their integration into existing recycling systems without disruption.

### Supplementary Information

Supplementary Information.

## Data Availability

The datasets used and analysed during the current study available from the corresponding author on reasonable request.

## References

[1] OECD. *Driving low-carbon innovations for climate neutrality*, https://www.oecd.org/publications/driving-low-carbon-innovations-for-climate-neutrality-8e6ae16b-en.htm (2023).

[2] OECD. *Climate change and plastics pollution*, https://www.oecd.org/environment/plastics/Policy-Highlights-Climate-change-and-plastics-pollution-Synergies-between-two-crucial-environmental-challenges.pdf (2023).

[3] OECD. *Plastic leakage and greenhouse gas emissions are increasing*, https://www.oecd.org/environment/plastics/increased-plastic-leakage-and-greenhouse-gas-emissions.htm (2020).

[4] Plastics Europe. *Plastics-the fast facts 2023*, https://plasticseurope.org/knowledge-hub/plastics-the-fast-facts-2023/ (2023).

[5] European Commission. *Biobased, biodegradable and compostable plastics*, https://environment.ec.europa.eu/topics/plastics/biobased-biodegradable-and-compostable-plastics_en#what-are-biobased-biodegradable-and-compostable-plastics (2020).

[6] OECD. *Environment Ministers’ commitments on plastics*, https://www.oecd.org/environment/ministerial/outcomes/Environment-Ministers-commitments-on-plastics.pdf (2022).

[7] Ministry of the Environment (Japan). *Roadmap for Bioplastics Introduction—For the sustainable use of plastics*, https://www.env.go.jp/recycle/roadmap_for_bioplastics_introduction.html (2021).

[8] Plastics Europe. *Plastics—the Facts 2022*, https://plasticseurope.org/knowledge-hub/plastics-the-facts-2022/ (2022).

[9] Europeanbioplastics. *Bioplastics market development update 2023*, https://www.european-bioplastics.org/market/ (2023).

[10] Teixeira S, Eblagon KM, Miranda F, Pereira MFR, Figueiredo JL (2021). Towards controlled degradation of poly(lactic) acid in technical applications. C J. Carbon Res..

[11] Slezak R, Krzystek L, Puchalski M, Krucińska I, Sitarski A (2023). Degradation of bio-based film plastics in soil under natural conditions. Sci. Total Environ..

[12] Delacuvellerie A (2021). Microbial biofilm composition and polymer degradation of compostable and non-compostable plastics immersed in the marine environment. J. Hazard. Mater..

[13] Fojt J, David J, Přikryl R, Řezáčová V, Kučerík J (2020). A critical review of the overlooked challenge of determining micro-bioplastics in soil. Sci. Total Environ..

[14] Piyathilake U (2024). Exploring the hidden environmental pollution of microplastics derived from bioplastics: A review. Chemosphere.

[15] Cucina M, de Nisi P, Tambone F, Adani F (2021). The role of waste management in reducing bioplastics’ leakage into the environment: A review. Bioresour. Technol..

[16] Wojnowska-Baryła I, Kulikowska D, Bernat K (2020). Effect of bio-based products on waste management. Sustainability.

[17] Kumagai S, Nakatani J, Saito Y, Fukushima Y, Yoshioka T (2020). Latest trends and challenges in feedstock recycling of polyolefinic plastics. J. Jpn. Pet. Inst..

[18] Kwon G, Cho D-W, Park J, Bhatnagar A, Song H (2023). A review of plastic pollution and their treatment technology: A circular economy platform by thermochemical pathway. Chem. Eng. J..

[19] Jahirul MI (2022). Transport fuel from waste plastics pyrolysis: A review on technologies, challenges and opportunities. Energy Convers. Manag..

[20] Kumagai S, Yoshioka T (2021). Chemical feedstock recovery from hard-to-recycle plastics through pyrolysis-based approaches and pyrolysis-gas chromatography. Bull. Chem. Soc. Jpn..

[21] Kumagai S, Yoshioka T (2016). Feedstock recycling via waste plastic pyrolysis. J. Jpn. Pet. Inst..

[22] Andooz A, Eqbalpour M, Kowsari E, Ramakrishna S, Ansari Cheshmeh Z (2023). A comprehensive review on pyrolysis from the circular economy point of view and its environmental and social effects. J. Clean. Prod..

[23] Jeswani H (2021). Life cycle environmental impacts of chemical recycling via pyrolysis of mixed plastic waste in comparison with mechanical recycling and energy recovery. Sci. Total Environ..

[24] Kopinke FD, Remmler M, Mackenzie K, Moeder M, Wachsen O (1996). Thermal decomposition of biodegradable polyesters. II. Poly(lactic acid). Polym. Degrad. Stab..

[25] Kopinke FD, Remmler M, Mackenzie K (1996). Thermal decomposition of biodegradable polyesters. I: poly(β-hydroxybutyric acid). Polym. Degrad. Stab..

[26] Shao Z, Kumagai S, Kameda T, Saito Y, Yoshioka T (2023). Effects of heating rate and temperature on product distribution of poly-lactic acid and poly-3-hydroxybutyrate-co-3-hydroxyhexanoate. J. Mater. Cycles Waste Manag..

[27] Saeaung K, Phusunti N, Phetwarotai W, Assabumrungrat S, Cheirsilp B (2021). Catalytic pyrolysis of petroleum-based and biodegradable plastic waste to obtain high-value chemicals. Waste Manag..

[28] Damayanti D (2022). Current prospects for plastic waste treatment. Polymers.

[29] Zhang Y (2023). A comprehensive review of separation technologies for waste plastics in urban mine. Resour. Conserv. Recycl..

[30] Sun C (2021). Exploring the synergetic effects of the major components of biomass additives in the pyrolysis of polylactic acid. Green Chem..

[31] Kasataka K, Kumagai S, Kameda T, Saito Y, Yoshioka T (2020). Enhancement of gasification and liquefaction during fast co-pyrolysis of cedar wood and polyethylene through control of synergistic interactions. Bioresour. Technol. Rep..

[32] Xie S, Kumagai S, Kameda T, Saito Y, Yoshioka T (2021). Prediction of pyrolyzate yields by response surface methodology: A case study of cellulose and polyethylene co-pyrolysis. Bioresour. Technol..

[33] Omura M, Tsukegi T, Shirai Y, Nishida H, Endo T (2006). Thermal degradation behavior of poly(lactic acid) in a blend with polyethylene. Ind. Eng. Chem. Res..

[34] Miskolczi N (2013). Co-pyrolysis of petroleum based waste HDPE, poly-lactic-acid biopolymer and organic waste. J. Ind. Eng. Chem..

[35] Wu X, Bourbigot S, Li K, Zou Y (2022). Co-pyrolysis characteristics and flammability of polylactic acid and acrylonitrile-butadiene-styrene plastic blend using TG, temperature-dependent FTIR, Py-GC/MS and cone calorimeter analyses. Fire Saf. J..

[36] Plastic Waste Management Institute. *An Introduction to Plastic Recycling 2023*, https://www.pwmi.or.jp/pdf/panf1.pdf (2023).

[37] Das P, Tiwari P (2017). Thermal degradation kinetics of plastics and model selection. Thermochim. Acta.

[38] Saad JM (2021). Comparison of waste plastics pyrolysis under nitrogen and carbon dioxide atmospheres: A thermogravimetric and kinetic study. J. Anal. Appl. Pyrol..

[39] Sun C (2021). Synergistic interaction for saving energy and promoting co-pyrolysis of polylactic acid and wood flour. Renew. Energy.

[40] Arrieta MP, Parres F, Lopez J, Jimenez A (2013). Development of a novel pyrolysis-gas chromatography/mass spectrometry method for the analysis of poly(lactic acid) thermal degradation products. J. Anal. Appl. Pyrol..

[41] Tsuge S, Hajima O, Watanabe C (2011). Pyrolysis-GC/MS Data Book of Synthetic Polymers.

