# Supplementary material for: Selective recovery of pyrolyzates of biodegradable (PLA, PHBH) and common plastics (HDPE, PP, PS) during co-pyrolysis under slow heating
Source: Sci Rep. 2024 Jul 16;14:16476. doi: 10.1038/s41598-024-67330-0 (PMC11252368; doi:10.1038/s41598-024-67330-0)
Supplement: Supplementary file 1 — Supplementary Information. [file 41598_2024_67330_MOESM1_ESM.docx]

Supporting information

Selective Recovery of Pyrolyzates of Biodegradable (PLA, PHBH) and Common Plastics (HDPE, PP, PS) During Co-pyrolysis Under Slow Heating

Wakana Adachi^1^, Shogo Kumagai^1,2 *^, Zhuze Shao^1^, Yuko Saito^1^, Toshiaki Yoshioka^1^

^1^ Graduate School of Environmental Studies, Tohoku University, 6-6-07 Aoba, Aramaki-aza, Aoba-ku, Sendai, Miyagi 980-8579, Japan

^2^ Graduate School of Engineering, Tohoku University, 6-6-07 Aoba, Aramaki-aza, Aoba-ku, Sendai, Miyagi 980-8579, Japan

^*^Corresponding author. E-mail address: kumagai@tohoku.ac.jp (S. Kumagai).

**Table S1** The summary of start, end, and peak-top temperatures of all the samples obtained by DTG

| Plastics | Temperature [°C] | | |
| --- | --- | --- | --- |
|  | *T*_s_^*1^ | *T*_f_^*2^ | *T*_p_^*3^ |
| PLA | 325 | 378 | 368 |
| PHBH | 273 | 298 | 294 |
| HDPE | 458 | 499 | 484 |
| PP | 422 | 478 | 465 |
| PS | 386 | 444 | 417 |
| ^*1^ Temperature at DTG = 5 wt%/min after weight loss start, ^*2^ Temperature at DTG = 5 wt%/min before weight loss finish, ^*3^ Temperature at the maximum DTG | | | |

**Table S2** The peak-top temperatures of all the samples obtained by EGA-MS (TIC)

| Samples | 1^st^ peak temperature [°C] | 2^nd^ peak temperature [°C] |
| --- | --- | --- |
| PLA | 364 |  |
| PHBH | 293 |  |
| HDPE | 478 |  |
| PP | 457 |  |
| PS | 411 |  |
| PLA10HDPE90 | 362 | 479 |
| PLA20HDPE80 | 357 | 476 |
| PLA50HDPE50 | 363 | 478 |
| PLA10PP90 | 356 | 457 |
| PLA20PP80 | 361 | 458 |
| PLA50PP50 | 362 | 457 |
| PLA10PS90 | 370 | 413 |
| PLA20PS80 | 370 | 414 |
| PLA50PS50 | 363 | 414 |
| PHBH10HDPE90 | 289 | 479 |
| PHBH20HDPE80 | 290 | 477 |
| PHBH50HDPE50 | 289 | 477 |
| PHBH10PP90 | 290 | 455 |
| PHBH20PP80 | 289 | 459 |
| PHBH50PP50 | 288 | 456 |
| PHBH10PS90 | 290 | 413 |
| PHBH20PS80 | 286 | 411 |
| PHBH50PS50 | 293 | 411 |


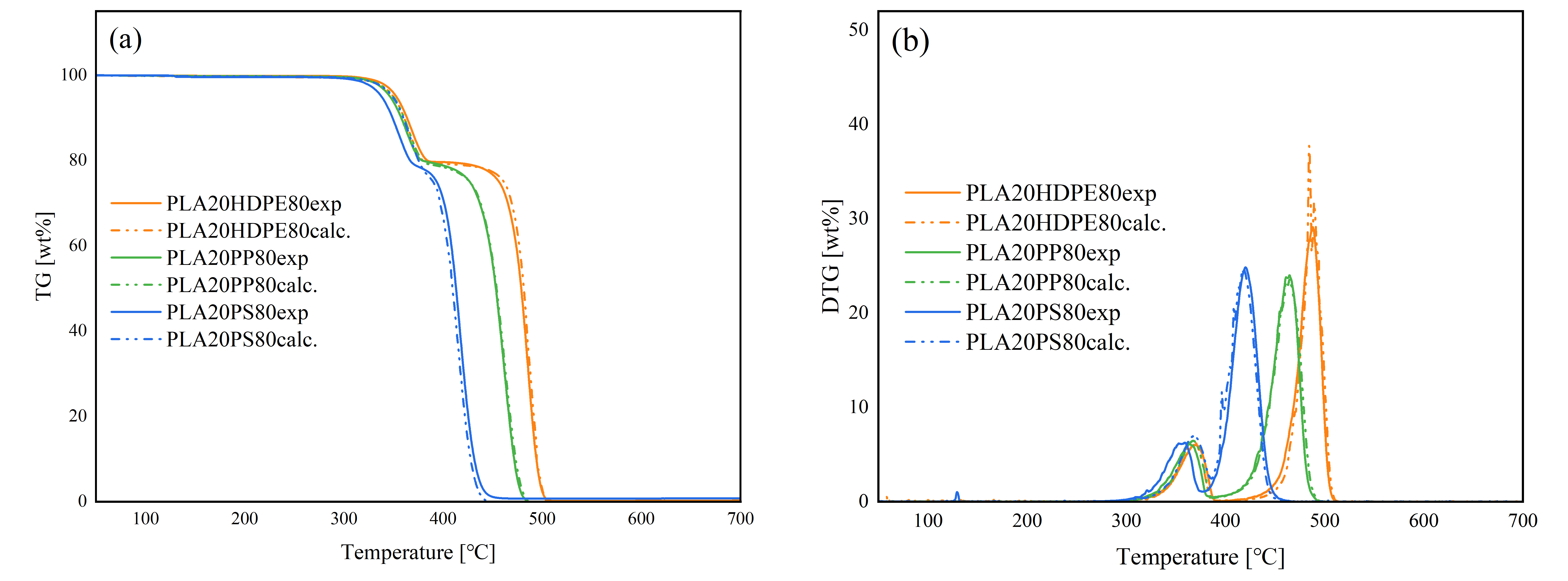


**Figure S1** TG (a) and DTG (b) curves of PLA20HDPE80, PLA20PP80, and PLA20PS80.


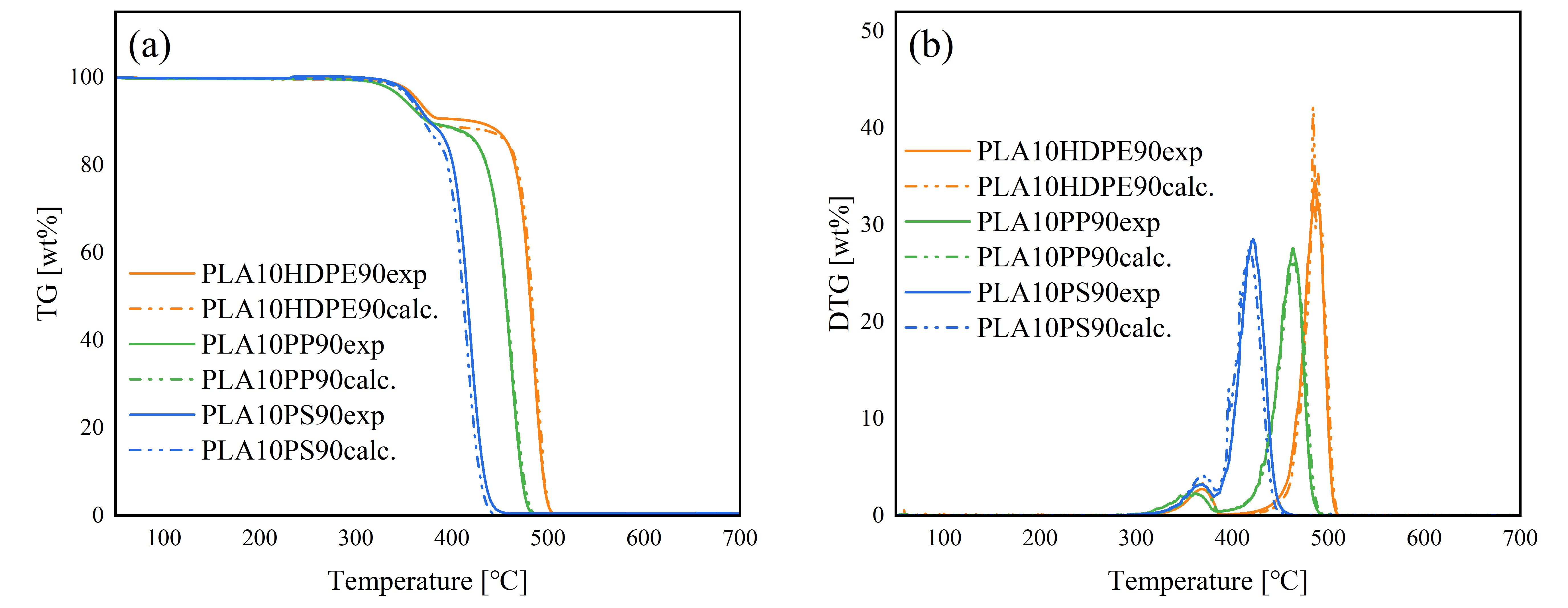


**Figure S2** TG (a) and DTG (b) curves of PLA10HDPE90, PLA10PP90, PLA10PS90


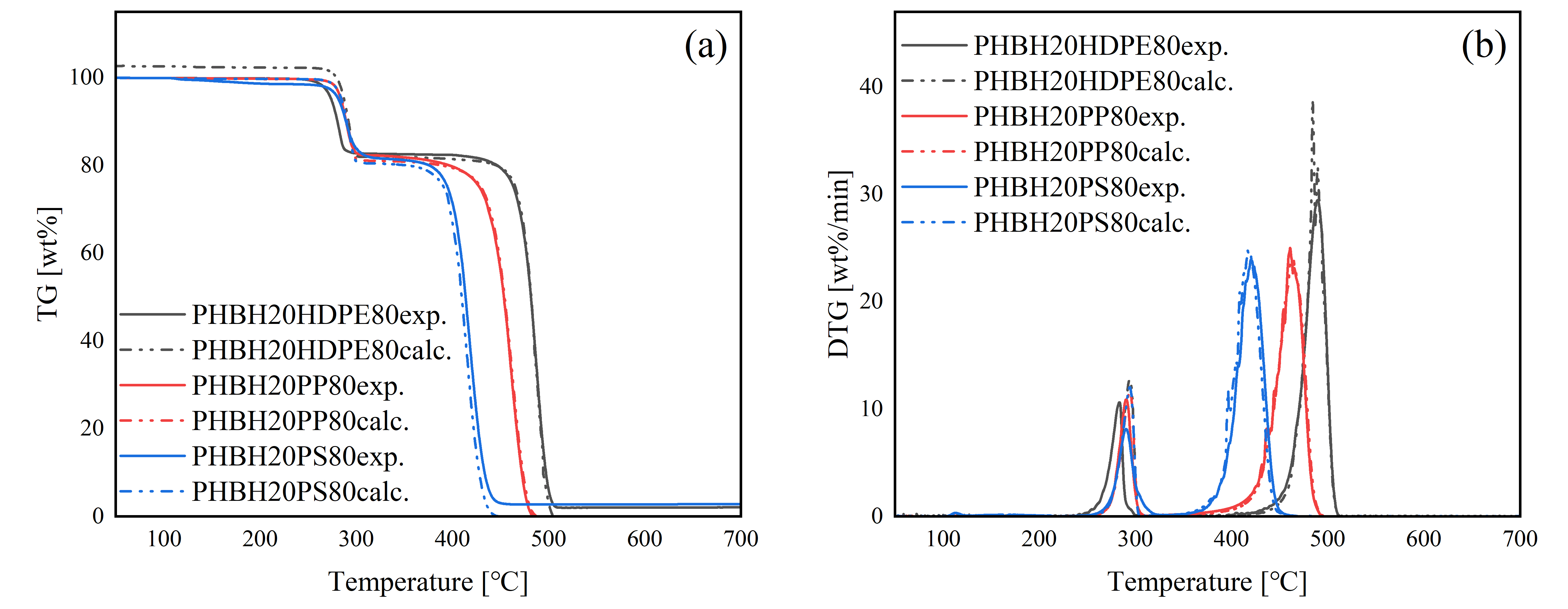


**Figure S3** TG (a) and DTG (b) curves of PHBH20HDPE80, PHBH20PP80, PHBH20PS80


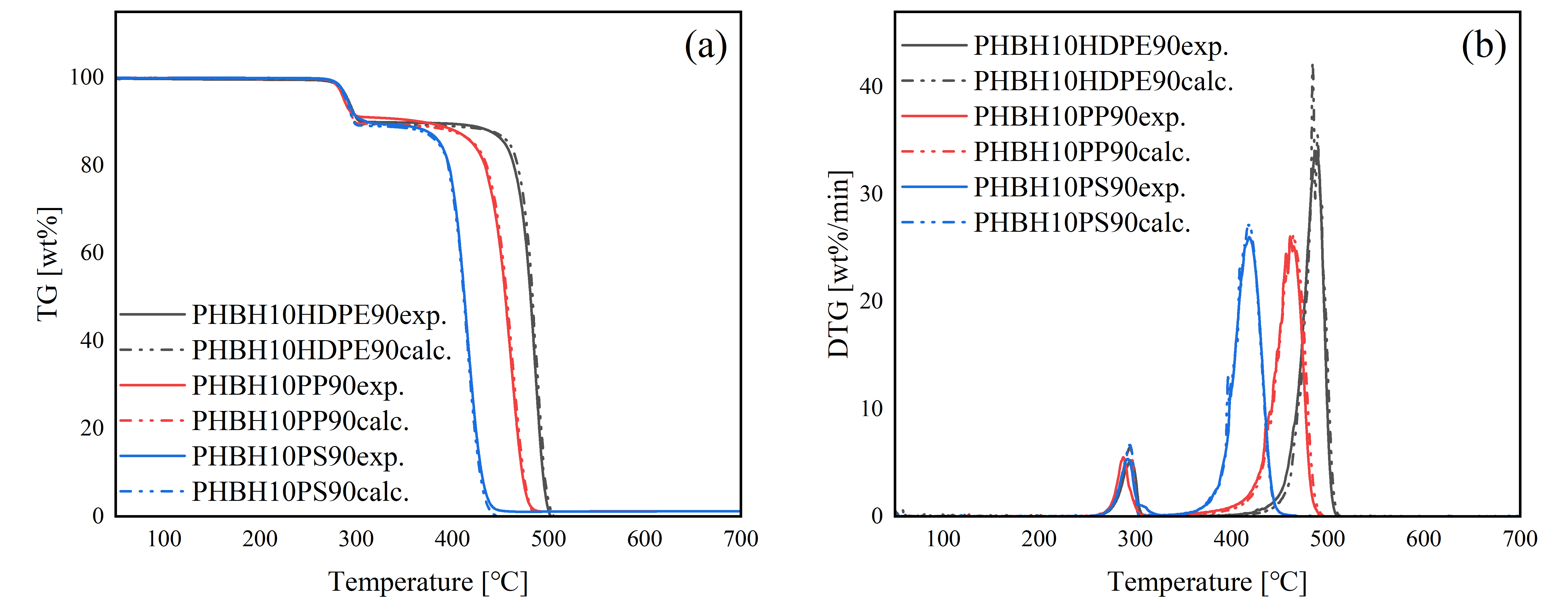


**Figure S4** TG (a) and DTG (b) curves··of PHBH10HDPE90, PHBH10PP90, PHBH10PS90


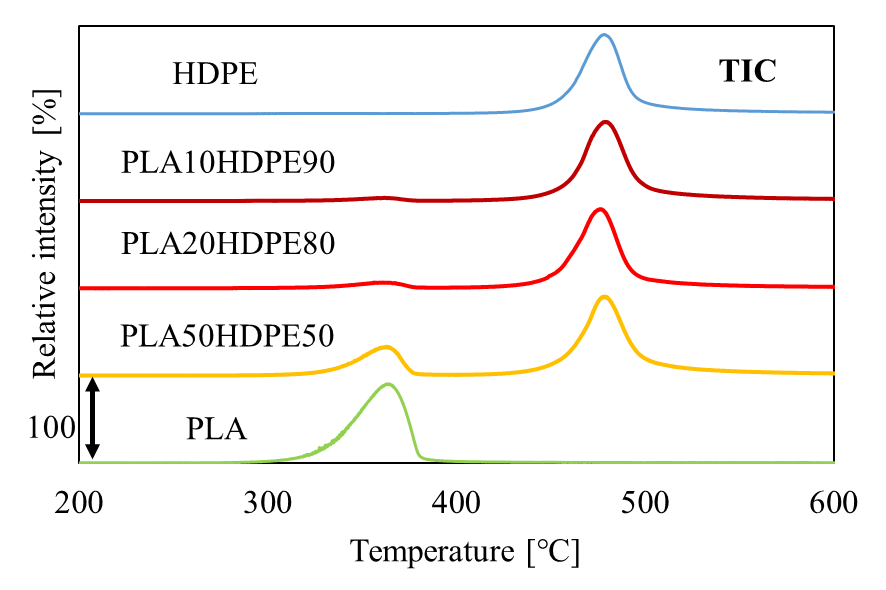


**Figure S5** TIC and EICs of PLA+HDPE


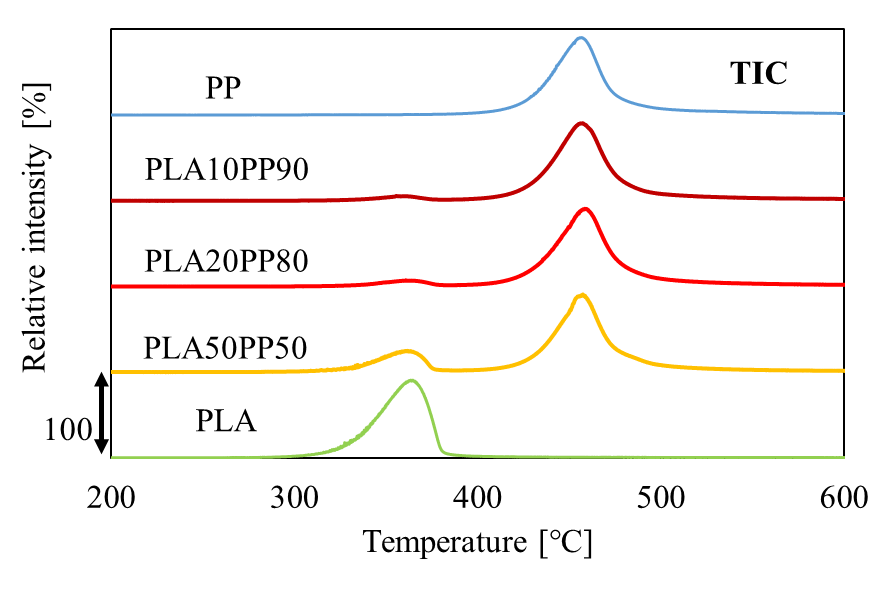


**Figure S6** TIC and EICs of PLA+PP


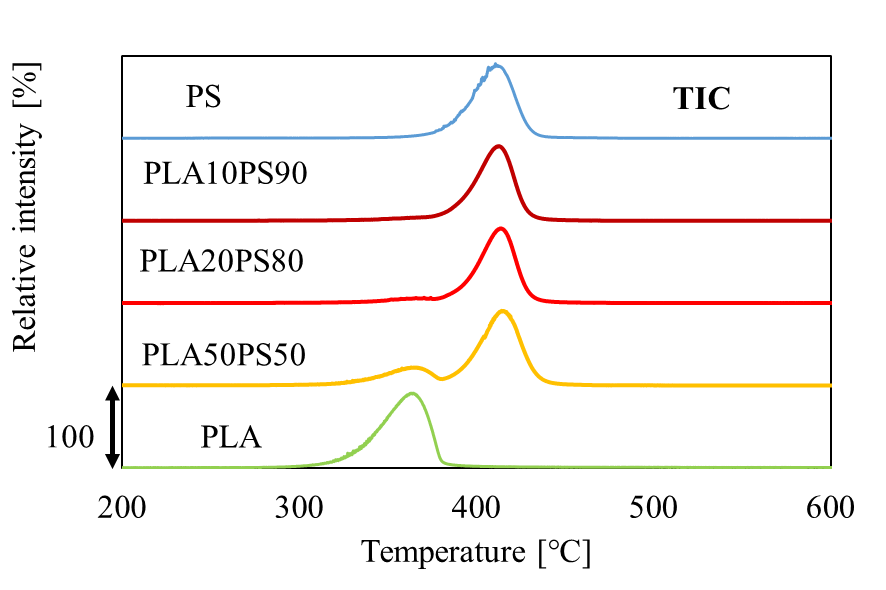


**Figure S7** TIC and EICs of PLA+PS


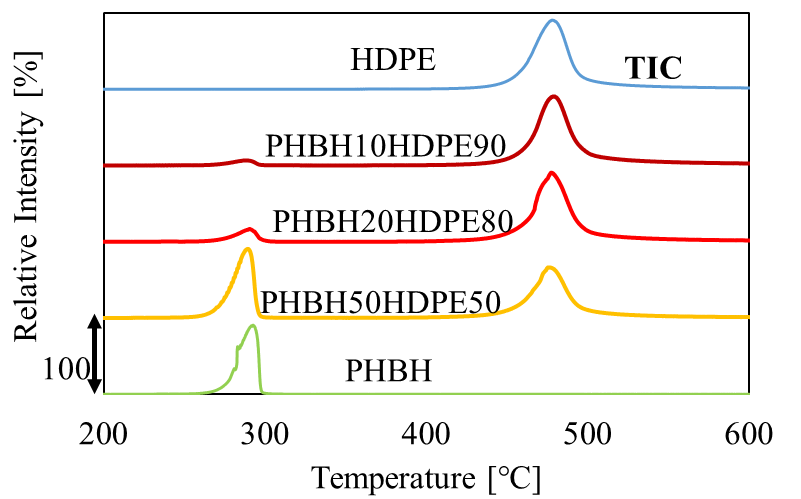


**Figure S8** TIC and EICs of PHBH+HDPE


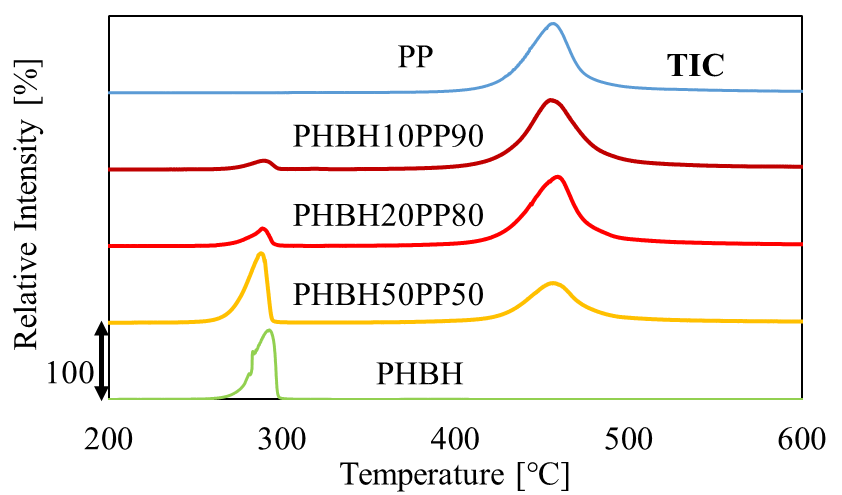


**Figure S9** TIC and EICs of PHBH+PP


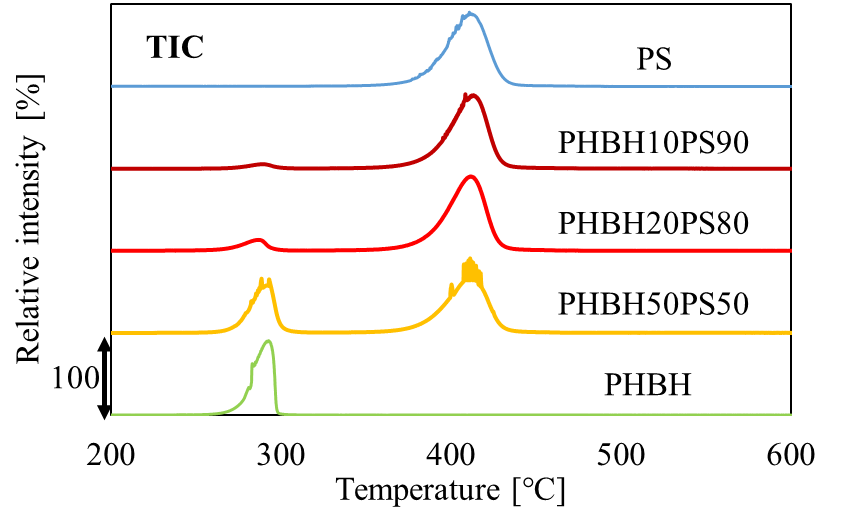


**Figure S10** TIC and EICs of PHBH+PS
